# Physicians’ Mutual Benefit Association of Texas

**Published:** 1888-12

**Authors:** 


F. E. DANIEL, M. D., EDITOR AND PUBLISHER.

THIS JOURNAL, by unanimous vote of the members, was made the Official Organ of the Austin District Medical Society.

COLLABORATORS:

*Dr. R. M. Swearingen, Austin.*
*Prof. B. E. Hadra, M. D., Galveston.*
*Prof. Geo. Cupples, M. D., San Antonio.*
*Dr. T. C. Osborn, Cleburne, Texas.*
*Dr E. J. Doering, Chicago.*
*Dr. E. J. Beall, Fort Worth.*
*Dr Odo Betz, Germany.*
*Dr. J. W. McLaughlin, Austin.*
*Dr. T. J. Tyner, Austin.*
*Prof. J. F. Y. Paine, M. D., Galveston.*
*Dr. R. H. L. Bibb, Mexico.*
*Dr. T. J. Bennett, Austin.*
*Dr. Bat Smith, Wharton.*
*Dr. E. Meierhof, New York.*
*Prof. W. B. Rogers, M. D., Memphis, Tenn.*

### PHYSICIANS' MUTUAL BENEFIT ASSOCIATION OF TEXAS.

President—W. A. Morris, M. D., Austin.

First Vice President—F. R. Martin, M. D., Kyle.

Second Vice President—M. A. Taylor, M. D., Austin.

Secretary and Treasurer—F. E. Daniel, M. D., Austin.

Board of Directors—R. M. Swearingin, M. D., Austin; J. W. McLaughlin, M. D., Austin; D. C. Hewson, M. D., Orange; S. A. Stone, M. D., Richmond; W. P. Burts, M. D., Fort Worth; Geo. Cupples, M. D., San Antonio; T. J. Bell, M. D., Tyler; R. H. Harrison, sr., M. D., Columbus.

Executive Committee—The President, Vice Presidents, Secretary, and Directors Swearingen, Cupples and McLaughlin.

The board of directors have the supervision of the business of the Order, and of the books of the secretary and treasurer; order the levying of assessments on the death of a member, and the payment of the proceeds to the beneficiary; assessments can be used for no other purpose.

At the Galveston meeting in April last, it was ordered that a circular letter to the profession of Texas be issued by the Board of Directors, calling on all reputable physicians to support the

Association; and in accordance therewith, the following is respectfully submitted:

*To the Medical Profession of Texas:*

In April, 1884, THE PHYSICIANS' MUTUAL BENEFIT ASSOCIATION OF TEXAS, was organized in Fort Worth, by a few zealous men, and chartered in June following. Its growth has been slow,—the membership the first two years not reaching one hundred—and to date the yearly average has been about one hundred and twenty. The plan of operation is very simple. It was organized upon the knowledge that many physicians spend their lives in the service of the sick, and utterly fail to provide for old age, or for their families after them; and that there are many physicians now, who by reason of poverty, old age or physical disability, cannot carry life insurance, and hence, at their death —their wives and children will be left destitute; upon the assumption also that there is not a physician *worthy of the name*, who would not cheerfully give *a dollar* to the widow or orphans of a deceased brother who has left them destitute,—if called on so to do.

The purpose of this organization therefore is, to *make that dollar available* to the widows and orphans of deceased members! to make it somebody's business to collect it, and to pay it over to such beneficiaries.

The organization is simple and its working uncomplicated. On the death of a member, the Secretary, under order of the Board of Directors, issues a call (assessment of one dollar, no more, no less,) upon the members, and each sends him one dollar, taking a receipt. This is deposited in the bank, and when all have paid, the aggregate sum (less the postage and printing) is sent to the person who was named in the deceased member's application as his beneficiary; sent as a free will offering, a *present* from the Society. A tax is provided of one dollar on joining, and annually thereafter, on the 1st of January—out of the aggregate of which all other expenses are paid, and *what is left*, if anything, goes to remunerate the Secretary for his services, he being the only officer who does any work, or receives any compensation whatever.

Thus it will be seen that the total cost of membership is one dollar per year, and there is no other expense, except in case of the death of a member, when each one is called upon for the pitiful sum of one dollar to make up a purse for the widow and children; can anything be simpler?

Now, as to the practical workings of it: In four years and eight months, (since organization to date,) there have been eight deaths only,—less than two a year,—out of an average membership of one hundred and twenty. In that time there has been paid to the eight widows, nine hundred and twenty dollars, an average of one hundred and fifteen dollars each; and to do this, it has cost the *oldest members* only one dollar and a half to each widow,—to-wit, four years' dues at $1, $4; eight assessments at $1, $8; total $12 in four years (not counting the fractional eight months,—in other words, less than three dollars a year.) With this pitiful tax, amounting to not exceeding twenty cents per month to those who have been members from the beginning, and too little to estimate, to those who have recently joined, the association has been enabled to collect and disburse over nine hundred dollars to the widows of eight of our deceased brothers; much good in a small way.

Experience has thus demonstrated the perfect feasibility of the plan.

Now, suppose there had been a thousand members instead of one hundred? The widows would have received one thousand dollars instead of one hundred dollars, and no member, not even the oldest, (in years or in date of membership) would have *felt* the cost of it:

Members of the Texas medical profession, where is your manhood? Your accredited charity and large philantrhopy? Were we, the organizers, *wrong* in assuming that "there is not a Texas physician *worthy of the name* who would refuse if called on, to give the destitute widow of a deceased brother a single dollar?" Have we over-estimated you? Do you care so little what becomes of your own wife and children after your death, as not to feel for those of the more unfortunate than you?

It will cost you only a single dollar to become an honored member of this truly charitable and benevolent little band, and experience has proven that the mortality amongst us is no greater than with others, and in future are not likely to be taxed by a heavy death rate. But suppose with a membership of 1,000, the death rate should be in the same ratio, it would not cost you exceeding $15 a year to safely carry an insurance of $1,000, which is much cheaper than any known reliable insurance can be effected.

Come to the support of this effort on the part of your brethren to provide a legacy of at least a thousand dollars for your loved ones when you are gone. It is only a matter of short time when you *will be* taken from them, and if you have made no other provision for them, make this at least. But, do not take a selfish view of it, and say "I have provided," or "I have insurance enough." Remember, there are *many* who have not; and our experience has demonstrated that the little gift of $115 in *three cases*, was a perfect Godsend—the widows being left destitute, and dependent on their labor for a living; or, on charity! Rally to the little nucleus; kept alive nearly five years, almost solely by the efforts of one man—so earnest is the desire to see the organization live and reach that stage where the widow will get a thousand, instead of a hundred dollars. Join now; send in your name, and that of your beneficiary, and *one dollar* to our secretary, and thus enroll your name not only in the order, but in the skies,---for "Ye that doeth it unto the least of one of these doeth it unto me."

By order of the Board of Directors.

